# Supplementary material for: Comparative Analysis of Structural Composition and Function of Intestinal Microbiota between Chinese Indigenous Laiwu Pigs and Commercial DLY Pigs
Source: Vet Sci. 2023 Aug 16;10(8):524. doi: 10.3390/vetsci10080524 (PMC10458769; doi:10.3390/vetsci10080524)
Supplement: Supplementary file 1 [file vetsci-10-00524-s001.zip › FIGURE&TABLE/Table S1.pdf]

Table S1: Sample information of Laiwu and DLY pig used in the study												
	Laiwu						DLY					
	LW1	LW2	LW3	LW4	LW5	LW6	DLY1	DLY2	DLY3	DLY4	DLY5	DLY6
Sex	male	male	male	male	male	male	male	male	male	male	male	male
Body weight(Kg)	85. 4	84. 86	89. 43	82. 71	87. 71	92. 43	129. 64	138. 21	130. 7	117. 3	118. 39	113. 04
