# Supplementary material for: Comparative Analysis of Structural Composition and Function of Intestinal Microbiota between Chinese Indigenous Laiwu Pigs and Commercial DLY Pigs
Source: Vet Sci. 2023 Aug 16;10(8):524. doi: 10.3390/vetsci10080524 (PMC10458769; doi:10.3390/vetsci10080524)
Supplement: Supplementary file 1 [file vetsci-10-00524-s001.zip › FIGURE&TABLE/Table S2.pdf]

Table S2: Summary statistics for the 16S rDNA-Seq

Sample	Sample Name	Raw PE(#)	Raw Tags(#)	Clean Tags(#)	Effective Tags(#)	Base(nt)	Avg Len(	Q20	Q30	GC%	Effective%
LW1	LWDU1	103,715	88,859	86,462	61,720	25,678,870	416	98.51	95.21	53.73	59.51
	LWJE1	76,891	55,162	50,669	45,487	18,361,450	404	98.64	95.43	52.77	59.16
	LWIL1	104,978	95,407	93,116	64,638	26,516,443	410	98.6	95.29	53.52	61.57
	LWCE1	81,514	69,173	67,550	52,932	21,329,996	403	98.47	94.99	52.76	64.94
	LWCO1	75,276	54,251	52,903	47,568	17,682,185	372	98.15	94.3	52.34	63.19
	LWRE1	104,504	89,670	86,529	62,441	25,800,361	413	98.31	94.55	52.44	59.75
LW2	LWDU2	108,749	94,376	91,968	67,610	28,172,515	417	98.35	94.78	53.35	62.17
	LWJE2	100,627	83,371	79,592	67,531	27,414,754	406	98.69	95.53	52.77	67.11
	LWIL2	104,831	93,527	90,934	67,723	28,007,489	414	98.56	95.34	54.13	64.6
	LWCE2	97,388	90,969	88,774	57,956	23,765,701	410	98.3	94.49	52.61	59.51
	LWCO2	86,916	72,497	71,643	50,846	20,475,489	403	98.48	95.03	52.91	58.5
	LWRE2	105,931	100,540	97,902	64,315	26,716,829	415	98.22	94.31	52.65	60.71
LW3	LWDU3	100,178	84,836	82,603	62,883	25,802,653	410	98.79	95.98	53.34	62.77
	LWJE3	105,393	80,489	79,376	62,278	25,901,195	416	98.46	94.91	53.38	59.09
	LWIL3	102,780	88,576	86,095	65,870	27,579,249	419	98.68	95.65	54.23	64.09
	LWCE3	97,168	93,254	91,256	58,536	24,026,559	410	98.37	94.76	52.65	60.24
	LWCO3	85,998	73,218	71,131	52,371	21,366,769	408	98.54	95.15	52.93	60.9
	LWRE3	97,565	82,297	79,688	62,124	25,499,723	410	98.37	94.69	52.8	63.67
LW4	LWDU4	99,839	88,688	87,160	64,654	26,706,668	413	98.57	95.3	53.93	64.76
	LWJE4	78,879	64,606	63,455	49,703	20,384,169	410	98.36	94.82	53.74	63.01
	LWIL4	113,481	98,496	95,766	67,494	27,997,355	415	98.53	95.11	54.19	59.48
	LWCE4	100,291	82,610	79,796	58,775	23,977,654	408	98.53	95.09	52.89	58.6
	LWCO4	102,331	84,747	83,689	60,922	25,072,093	412	98.49	95.04	53.19	59.53
	LWRE4	97,757	89,068	88,256	62,233	25,469,684	409	98.38	94.78	52.71	63.66
LW5	LWDU5	100,055	96,319	95,112	66,409	26,944,777	406	98.83	95.9	53.49	66.37
	LWJE5	104,762	80,608	76,692	65,119	26,734,576	411	98.65	95.37	53.61	62.16
	LWIL5	102,235	92,221	89,889	64,890	27,101,952	418	98.48	95.05	53.68	63.47
	LWCE5	105,697	94,655	91,597	62,667	25,641,007	409	98.47	95.01	52.75	59.29
	LWCO5	93,348	71,458	69,678	54,648	22,202,559	406	98.53	95.12	52.92	58.54
	LWRE5	105,085	90,720	88,231	63,344	25,871,988	408	98.53	95.11	52.88	60.28
LW6	LWDU6	101,211	86,572	84,424	61,413	25,466,503	415	98.1	94.11	53.6	60.68
	LWJE6	103,503	97,559	94,701	65,424	26,761,275	409	97.74	93.07	53.24	63.21
	LWIL6	100,202	75,615	72,197	61,643	25,547,943	414	98.63	95.34	53.71	61.52
	LWCE6	100,511	96,964	95,103	62,738	25,858,545	412	98.43	94.81	52.33	62.42
	LWCO6	83,208	81,552	80,558	49,198	20,239,418	411	98.34	94.68	53.4	59.13
	LWRE6	77,959	62,215	60,165	50,221	20,319,098	405	98.37	94.69	52.56	64.42
DLY1	DLYDU1	98,859	84,669	82,788	62,960	26,134,827	415	98.49	95.21	53.72	63.69
	DLYJE1	109,497	91,569	89,001	69,217	28,345,308	410	98.32	94.74	53.64	63.21
	DLYIL1	104,273	88,609	85,577	61,796	25,310,114	410	98.63	95.54	53.74	59.26
	DLYCE1	104,244	87,140	84,862	65,128	26,756,691	411	98.39	94.76	52.61	62.48
	DLYCO1	98,949	95,589	93,789	62,094	25,545,705	411	98.38	94.78	52.69	62.75
	DLYRE1	102,857	94,796	92,052	62,254	26,008,749	418	98.26	94.46	52.94	60.52
DLY2	DLYDU2	97,961	86,281	83,778	58,046	24,304,145	419	98.15	94.16	54.01	59.25
	DLYJE2	105,122	99,635	98,157	66,297	26,718,080	403	98.68	95.53	52.87	63.07
	DLYIL2	99,010	70,507	65,228	59,767	23,083,251	386	98.47	95.03	53	60.36
	DLYCE2	110,995	89,337	86,581	65,784	26,990,675	410	98.33	94.64	52.71	59.27
	DLYCO2	102,321	91,610	89,211	63,786	26,533,038	416	98.31	94.61	53.34	62.34
	DLYRE2	97,159	86,787	86,224	63,057	26,180,467	415	98.37	94.68	53.17	64.9
	DLYDU3	97,796	84,265	82,006	61,903	25,786,888	417	98.35	94.76	53.5	63.3

DLY3	DLYJE3	81,568	55,361	52,427	49,346	19,296,238	391	98.61	95.4	52.81	60.5
	DLYIL3	105,497	83,566	79,401	62,206	25,011,336	402	98.51	95.09	52.98	58.96
	DLYCE3	97,908	81,667	78,556	61,874	25,184,826	407	98.34	94.7	52.83	63.2
	DLYCO3	91,943	77,943	76,467	58,413	23,791,547	407	98.5	95.03	53.08	63.53
	DLYRE3	109,843	98,564	96,260	65,050	26,602,565	409	98.47	95.01	52.94	59.22
DLY4	DLYDU4	104,330	91,324	89,027	64,203	26,828,441	418	98.33	94.71	53.66	61.54
	DLYIL4	91,263	73,587	70,831	58,236	23,499,771	404	98.67	95.49	53.06	63.81
	DLYCE4	97,663	77,213	73,799	57,807	23,569,777	408	98.29	94.55	52.73	59.19
	DLYCO4	89,702	79,883	77,613	54,582	22,666,271	415	98.32	94.61	52.75	60.85
	DLYRE4	95,689	84,608	82,031	59,672	24,835,304	416	98.38	94.76	53.48	62.36
DLY5	DLYDU5	115,435	97,724	94,251	69,545	29,228,792	420	98.25	94.52	53.37	60.25
	DLYJE5	102,436	81,648	78,298	60,696	24,854,073	409	98.6	95.26	53.27	59.25
	DLYIL5	105,667	92,484	89,648	62,246	25,855,201	415	98.7	95.73	53.98	58.91
	DLYCE5	74,757	57,481	56,215	46,892	18,900,424	403	98.35	94.68	52.7	62.73
	DLYCO5	106,516	99,172	96,167	64,201	27,117,262	422	98.09	94.01	51.58	60.27
	DLYRE5	97,108	90,279	87,816	62,343	26,227,622	421	98.12	94.13	52.76	64.2
DLY6	DLYDU6	103,745	88,025	85,612	62,711	26,432,056	421	98.33	94.78	53.05	60.45
	DLYJE6	97,416	87,732	85,653	60,414	25,331,092	419	98.61	95.27	53.64	62.02
	DLYIL6	88,682	62,488	55,571	55,169	19,960,594	362	98.32	94.83	52.78	62.21
	DLYCE6	109,069	92,020	90,669	67,770	27,431,853	405	98.26	94.49	52.68	62.13
	DLYCO6	101,532	89,885	87,367	61,614	25,427,053	413	98.52	95.08	52.65	60.68
	DLYRE6	97,596	90,798	88,462	62,031	25,871,353	417	98.36	94.71	54.07	63.56
