# Supplementary material for: Comparative Analysis of Structural Composition and Function of Intestinal Microbiota between Chinese Indigenous Laiwu Pigs and Commercial DLY Pigs
Source: Vet Sci. 2023 Aug 16;10(8):524. doi: 10.3390/vetsci10080524 (PMC10458769; doi:10.3390/vetsci10080524)
Supplement: Supplementary file 1 [file vetsci-10-00524-s001.zip › FIGURE&TABLE/Table S3.pdf]

**Table S3: Detail informations of the microbial community structure identified in the study**											
Taxonomy	Firmicutes	Proteobacteria	Spirochaetes	Bacteroidetes	Euryarchaeota	Actinobacteria	Fusobacteria	unidentified_Bacteria	Acidobacteria	Verrucomicrobia	Others
LWDU	77.67%	8.93%	0.07%	0.18%	0.00%	7.77%	0.10%	1.48%	0.77%	0.07%	2.95%
LWJE	75.81%	15.88%	0.05%	1.94%	0.06%	4.54%	0.10%	0.33%	0.07%	0.27%	0.96%
LWIL	56.13%	35.66%	0.03%	0.28%	0.02%	2.66%	0.70%	1.05%	0.58%	0.06%	2.84%
LWCE	81.62%	6.78%	0.35%	5.45%	0.06%	0.30%	1.74%	2.27%	0.00%	0.20%	1.21%
LWCO	69.43%	0.71%	8.47%	11.16%	4.75%	1.18%	0.03%	2.05%	0.00%	0.06%	2.16%
LWRE	66.56%	0.86%	8.49%	15.18%	4.18%	0.18%	0.13%	2.87%	0.01%	0.11%	1.42%
DLYDU	70.26%	7.67%	0.04%	0.19%	0.01%	12.87%	0.17%	2.83%	1.26%	0.13%	4.57%
DLYJE	82.57%	10.47%	0.15%	0.81%	0.05%	4.19%	0.09%	0.49%	0.07%	0.11%	1.01%
DLYIL	85.13%	7.48%	0.23%	0.56%	0.02%	1.72%	0.14%	0.83%	0.35%	0.03%	3.49%
DLYCE	89.63%	1.08%	0.66%	4.79%	0.08%	0.16%	0.01%	2.45%	0.01%	0.04%	1.11%
DLYCO	85.23%	0.94%	0.43%	9.88%	0.06%	0.22%	0.04%	2.41%	0.00%	0.02%	0.76%
DLYRE	63.60%	0.42%	3.87%	27.44%	0.21%	0.17%	0.02%	2.85%	0.00%	0.01%	1.41%
Taxonomy	Terrisporobacter	Clostridium_sensu_stricto 1	Lactobacillus	Romboutsia	Escherichia-Shigella	Treponema	Streptococcus	Turicibacter	Methanobrevibacter	UCG-002	Others
LWDU	19.09%	25.10%	8.95%	6.62%	6.50%	0.05%	3.03%	8.53%	0.00%	0.03%	22.11%
LWJE	47.60%	4.36%	3.66%	8.69%	10.45%	0.05%	3.93%	1.37%	0.05%	0.02%	19.82%
LWIL	16.55%	3.16%	4.87%	24.73%	33.07%	0.01%	0.88%	1.65%	0.01%	0.02%	15.05%
LWCE	31.14%	3.92%	0.95%	3.30%	2.19%	0.31%	0.54%	10.00%	0.06%	1.11%	46.48%
LWCO	16.21%	11.74%	2.17%	2.54%	0.42%	8.45%	0.77%	7.97%	4.75%	3.41%	41.57%
LWRE	14.72%	3.53%	1.49%	1.09%	0.40%	8.07%	0.95%	5.74%	4.18%	6.63%	53.21%
DLYDU	9.48%	10.13%	20.42%	4.84%	2.35%	0.00%	11.34%	2.64%	0.01%	0.02%	38.79%
DLYJE	45.37%	15.02%	1.15%	5.57%	8.65%	0.15%	2.08%	1.61%	0.04%	0.03%	20.32%
DLYIL	34.91%	15.22%	1.13%	24.49%	6.29%	0.22%	0.58%	3.28%	0.02%	0.12%	13.73%
DLYCE	32.50%	4.39%	0.26%	2.83%	0.54%	0.62%	0.81%	19.80%	0.06%	1.67%	36.52%
DLYCO	15.64%	8.30%	15.37%	1.45%	0.74%	0.40%	15.50%	2.36%	0.03%	4.58%	35.62%
DLYRE	6.32%	4.53%	7.80%	0.48%	0.28%	3.43%	12.31%	0.98%	0.17%	6.78%	56.93%
