# Supplementary figures and images for: Comparative Analysis of Structural Composition and Function of Intestinal Microbiota between Chinese Indigenous Laiwu Pigs and Commercial DLY Pigs
Source: Vet Sci. 2023 Aug 16;10(8):524. doi: 10.3390/vetsci10080524 (PMC10458769; doi:10.3390/vetsci10080524)

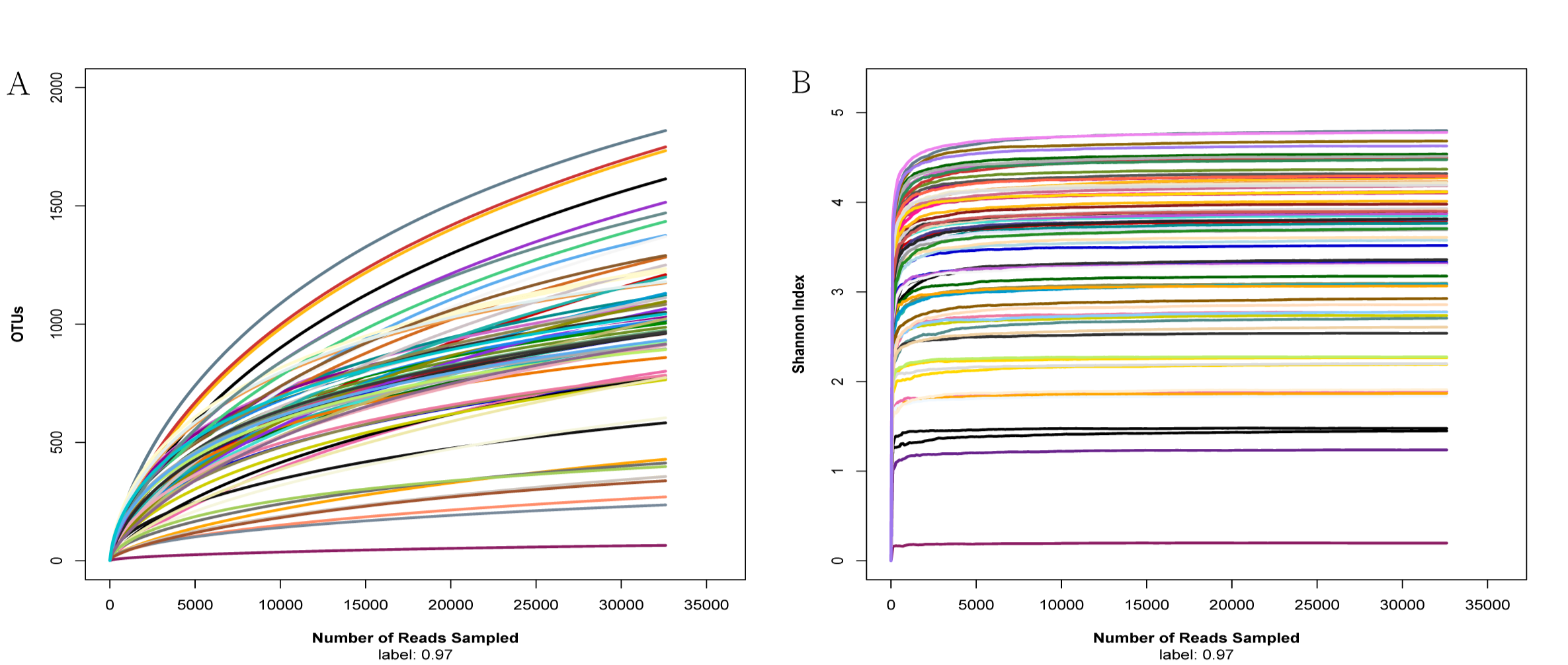

Supplement: Supplementary file 1 [file vetsci-10-00524-s001.zip › FIGURE&TABLE/Figure 1.tif]

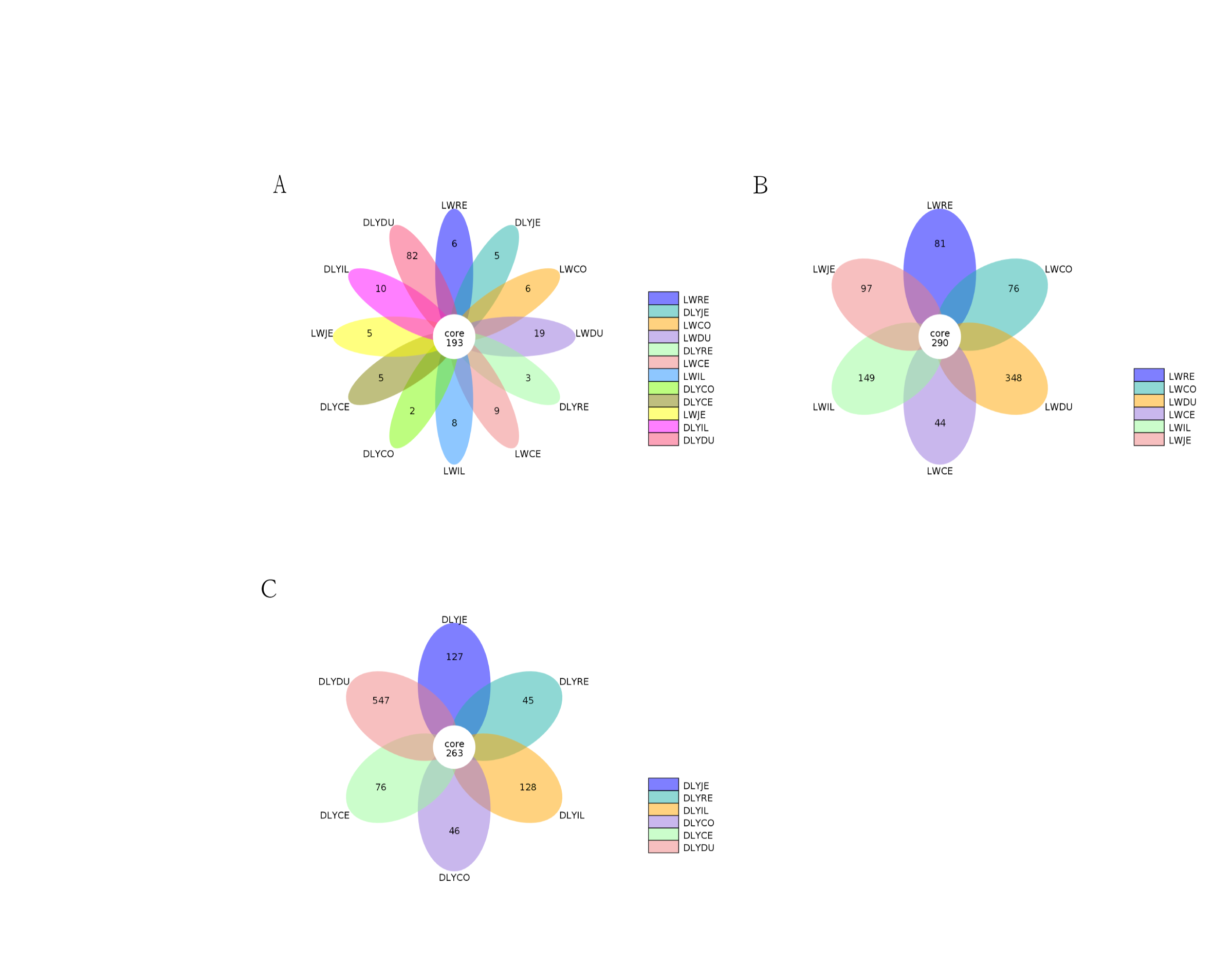

Supplement: Supplementary file 1 [file vetsci-10-00524-s001.zip › FIGURE&TABLE/Figure 2.tif]

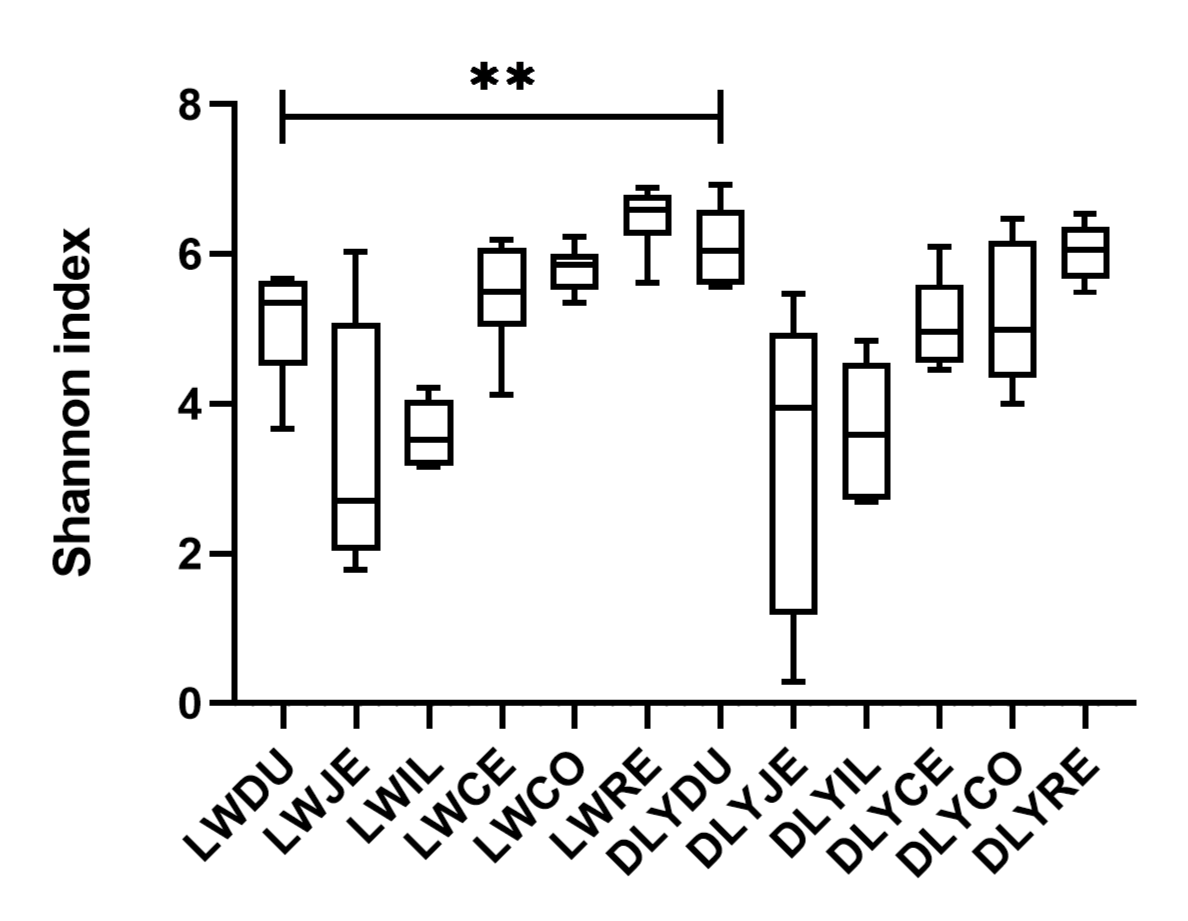

Supplement: Supplementary file 1 [file vetsci-10-00524-s001.zip › FIGURE&TABLE/Figure 3.tif]

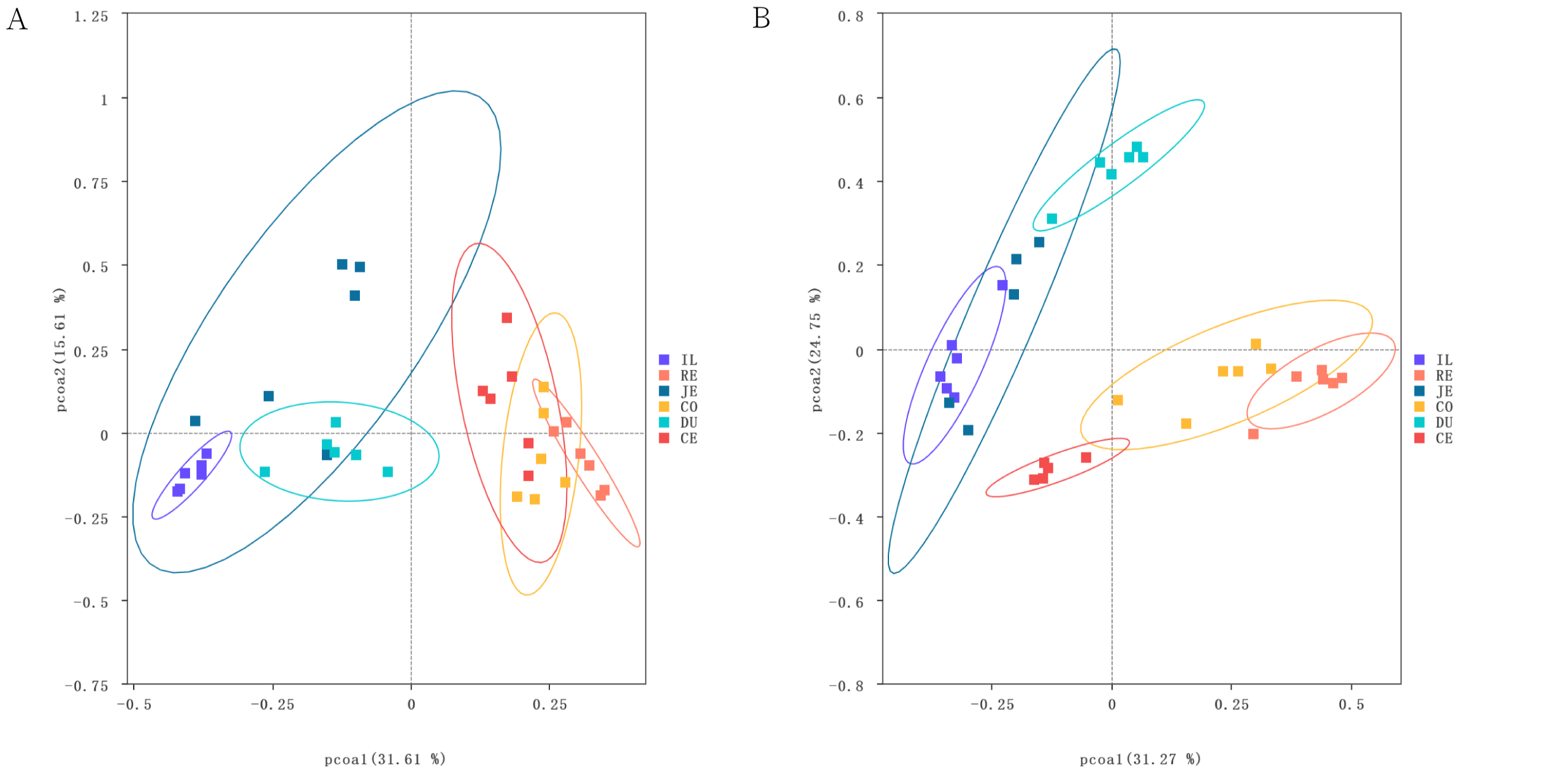

Supplement: Supplementary file 1 [file vetsci-10-00524-s001.zip › FIGURE&TABLE/Figure 4.tif]

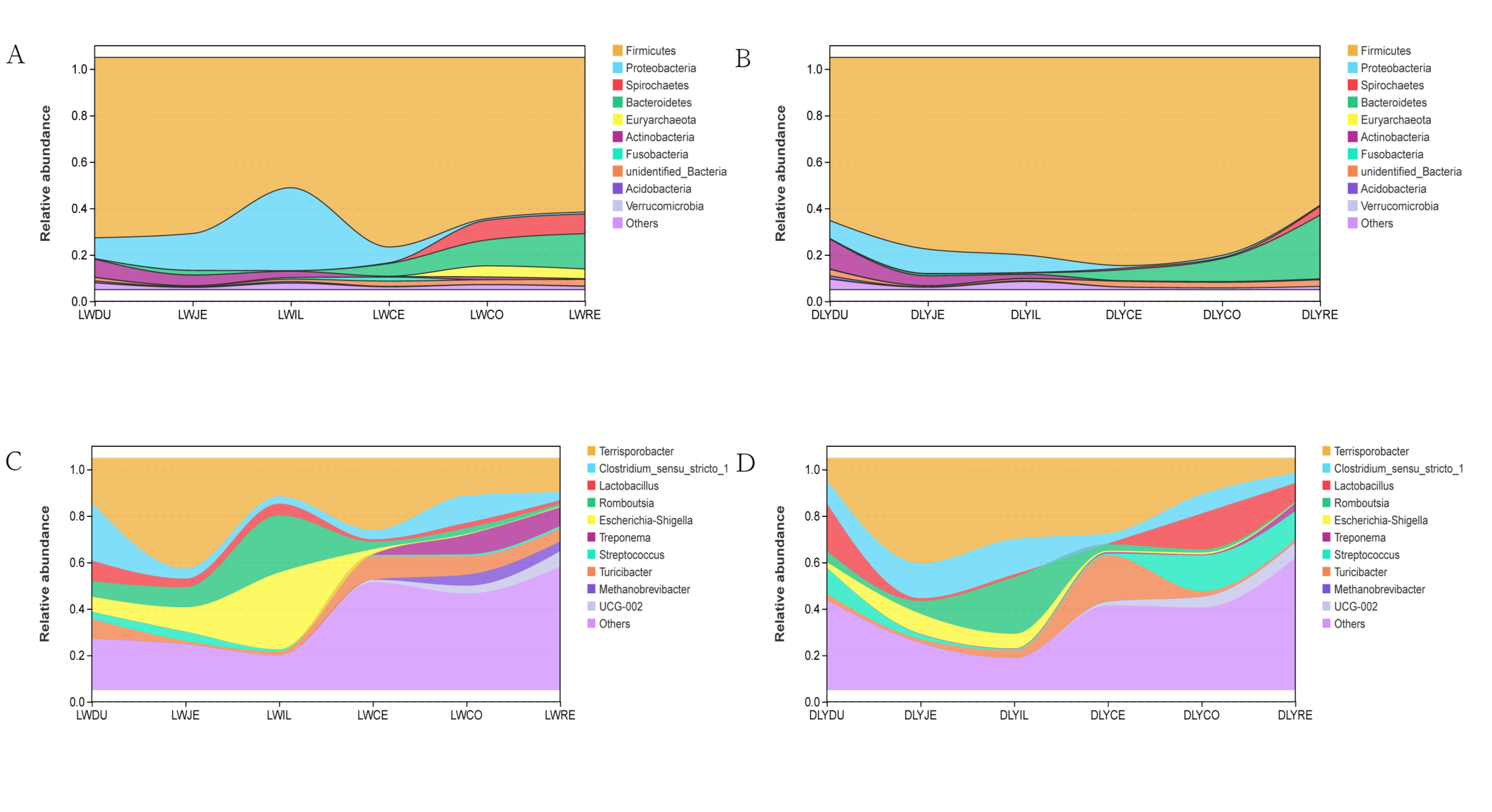

Supplement: Supplementary file 1 [file vetsci-10-00524-s001.zip › FIGURE&TABLE/Figure 5.tif]

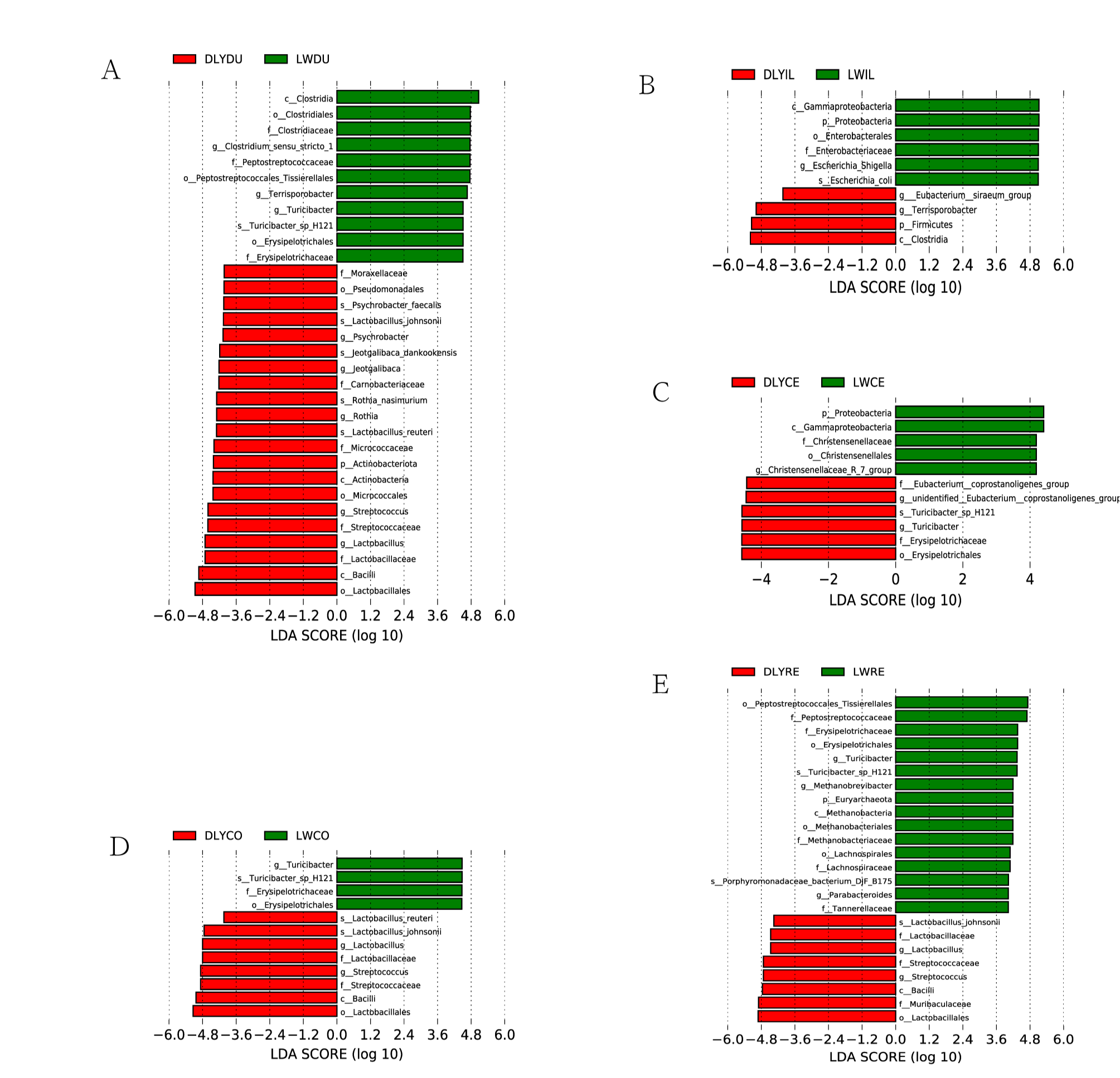

Supplement: Supplementary file 1 [file vetsci-10-00524-s001.zip › FIGURE&TABLE/Figure 6.tif]

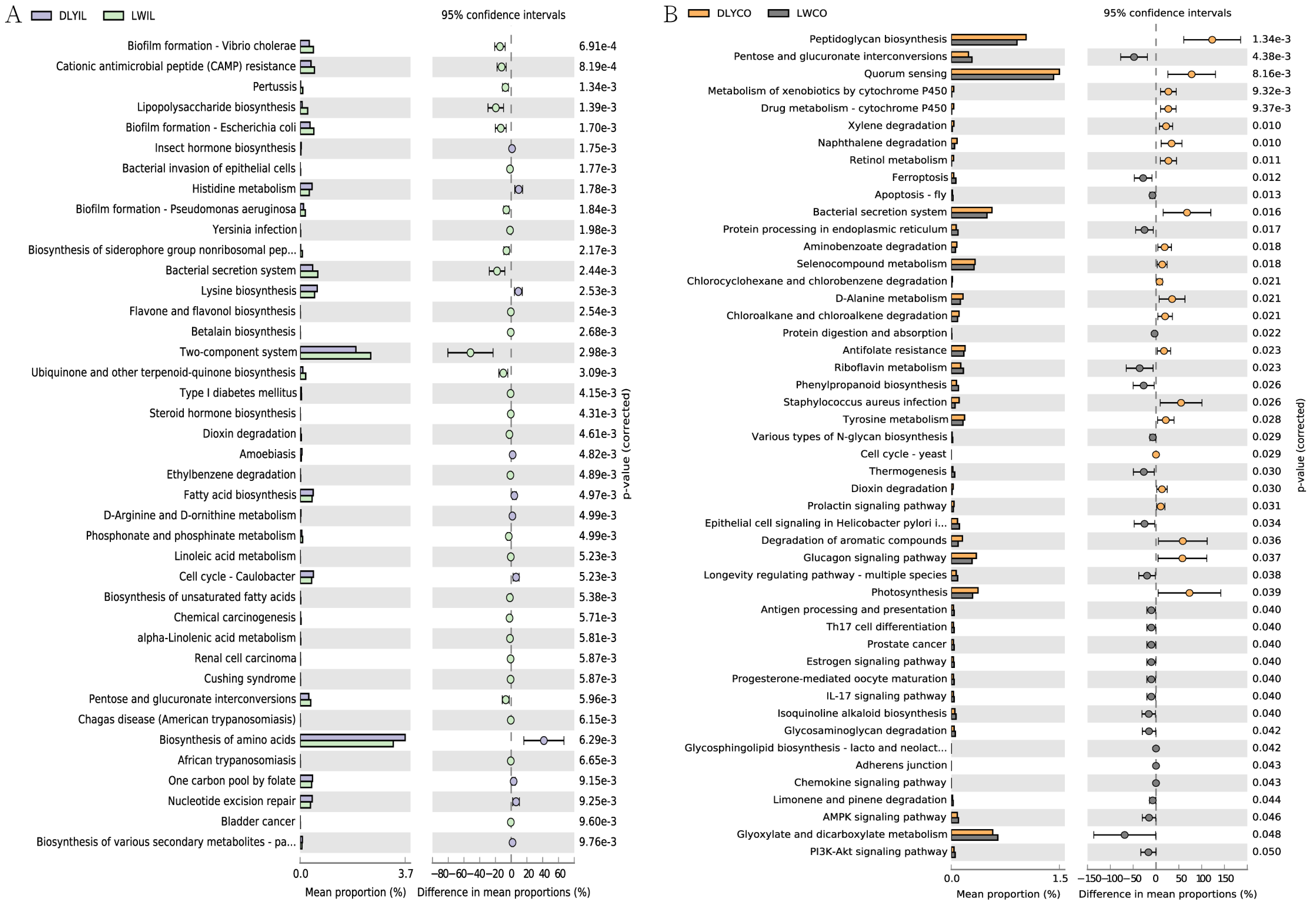

Supplement: Supplementary file 1 [file vetsci-10-00524-s001.zip › FIGURE&TABLE/Figure 7.tif]
